# Single nucleotide variants and InDels identified from whole-genome re-sequencing of Guzerat, Gyr, Girolando and Holstein cattle breeds

**DOI:** 10.1371/journal.pone.0173954

**Published:** 2017-03-21

**Authors:** Nedenia Bonvino Stafuzza, Adhemar Zerlotini, Francisco Pereira Lobo, Michel Eduardo Beleza Yamagishi, Tatiane Cristina Seleguim Chud, Alexandre Rodrigues Caetano, Danísio Prado Munari, Dorian J. Garrick, Marco Antonio Machado, Marta Fonseca Martins, Maria Raquel Carvalho, John Bruce Cole, Marcos Vinicius Gualberto Barbosa da Silva

**Affiliations:** 1 Departamento de Ciências Exatas, Universidade Estadual Paulista, Faculdade de Ciências Agrárias e Veterinárias, Jaboticabal, São Paulo, Brazil; 2 Embrapa Informática Agropecuária, Campinas, São Paulo, Brazil; 3 Embrapa Recursos Genéticos e Biotecnologia, Brasília, Distrito Federal, Brazil; 4 Department of Animal Science, Iowa State University, Ames, Iowa, United States of America; 5 Embrapa Gado de Leite, Juiz de Fora, Minas Gerais, Brazil; 6 Departamento de Biologia Geral, Universidade Federal de Minas Gerais, Belo Horizonte, Minas Gerais, Brazil; 7 United States Department of Agriculture, Agricultural Research Service, Animal Genomics and Improvement Laboratory, Beltsville, Maryland, United States of America; Institute of Farm Animal Genetics, GERMANY

## Abstract

Whole-genome re-sequencing, alignment and annotation analyses were undertaken for 12 sires representing four important cattle breeds in Brazil: Guzerat (multi-purpose), Gyr, Girolando and Holstein (dairy production). A total of approximately 4.3 billion reads from an Illumina HiSeq 2000 sequencer generated for each animal 10.7 to 16.4-fold genome coverage. A total of 27,441,279 single nucleotide variations (SNVs) and 3,828,041 insertions/deletions (InDels) were detected in the samples, of which 2,557,670 SNVs and 883,219 InDels were novel. The submission of these genetic variants to the dbSNP database significantly increased the number of known variants, particularly for the indicine genome. The concordance rate between genotypes obtained using the Bovine HD BeadChip array and the same variants identified by sequencing was about 99.05%. The annotation of variants identified numerous non-synonymous SNVs and frameshift InDels which could affect phenotypic variation. Functional enrichment analysis was performed and revealed that variants in the olfactory transduction pathway was over represented in all four cattle breeds, while the ECM-receptor interaction pathway was over represented in Girolando and Guzerat breeds, the ABC transporters pathway was over represented only in Holstein breed, and the metabolic pathways was over represented only in Gyr breed. The genetic variants discovered here provide a rich resource to help identify potential genomic markers and their associated molecular mechanisms that impact economically important traits for Gyr, Girolando, Guzerat and Holstein breeding programs.

## Introduction

Brazil has the world's largest commercial cattle herd with around 215.2 million head in 2015 according to the agricultural census [1], from which about 80% is estimated to be composed of purebred and crossbreed *Bos primigenius indicus* animals. The vast majority of Brazilian dairy cows are extensively grazed in challenging environments where they must convert low-quality forages into high-quality milk [2]. Due to the naturally occurring temperature, disease and nutritional stresses, indicine breeds are widely used in Brazilian cattle production systems, because of their adaptability towards the tropical climate and extensive production systems.

Guzerat and Gyr, which were introduced from India, are important indicine cattle breeds for milk and meat production in Brazil. The Guzerat breed was introduced in Brazil at the end of the 19^th^ century and currently ranks fifth in numbers of animals among indicine cattle breeds in the country [3]. The Guzerat breed is recognized for traits such as resistance to parasites, heat tolerance and willingness to consume forage with low nutritional value, all of which are important to its adaptation to adverse tropical environments. This breed was included in the FAO list of domestic animal genetic resources to be conserved by management [4] due to their potential for production in the tropics and their small effective population size.

Considered to be a dual-purpose breed, Guzerat cattle have been widely used in both purebred and crossbreeding schemes to produce efficient dairy cows and beef calves. The National Breeding Program for the Improvement of Guzerat Dairy Cattle was implemented in 1994 to improve milk production, conformation, and management traits through selection based on genetic evaluations through progeny testing and by utilizing multiple ovulation and embryo transfer nucleus schemes [5].

The Gyr breed is the indicine breed with the highest milk yield and it is recognized for its robustness, adaptability to high-temperature conditions and resistance to parasites common in tropical climates. The National Breeding Program of the Dairy Gyr was established in 1985 in Brazil aiming for the genetic improvement of the breed through the identification and selection of genetically superior bulls for milk production (milk, fat, protein and total solids), conformation and management traits [6]. Gyr animals are present in more than 80% of Brazilian dairy herds either as purebreds or in stabilized crosses with Holstein (*Bos primigenius taurus*) resulting in the Girolando composite breed [7]. Crossbreeding has been used to generate cows that would combine the high milk production capacity of Holsteins and the adaptability to tropical conditions of the Gyr breed. Crossbreds Girolando cattle are noteworthy for excellent productivity, high fertility indexes and good vigor. Due to these advantages, Girolando became the predominant cattle breed on the majority of Brazilian dairy farms in terms of numbers of animals.

A considerable number of genetic variants, including single nucleotide variations (SNVs), insertions/deletions (InDels), and structural variations have been identified across the cattle genome, as a result of the Bovine Genome Sequencing [8], HapMap [9], and the 1000 bull genomes projects [10]. These projects have helped to demonstrate the potential of cattle genomic research by increasing our knowledge of mammalian evolution and biology. Recent advances in next-generation sequencing (NGS) technology and sequence analysis tools have allowed whole genome re-sequencing to become a feasible tool to quickly, efficiently and accurately identify genetic variants segregating in a population. The identification of all genetic variants in the genome is a crucial first step for discovery of causal variants associated with complex traits in livestock species [11, 12]. Whole-genome re-sequencing has now been extensively used to identify genomic variants in a number of cattle breeds [13–21]. However, most studies have concentrated on taurine breeds, exacerbating the deficiency of knowledge for indicine genetic resources.

About 99.71 million single nucleotide variants (SNV) and 8.36 million InDels across the bovine genome have as of February 2017 been published in the dbSNP database (http://www.ncbi.nlm.nih.gov/snp/). Among these, only 4.38 million SNPs and three InDels have been deposited for indicine breeds. Furthermore, the SNP chips currently used for genotyping are biased toward variants useful for characterizing taurine breeds, imposing limitations for genome-wide studies about genetic structure and diversity of indicine breeds [22]. This bias strongly affects the estimates obtained from the data [23–25]. Nevertheless, commercially available SNP chips of low- and medium-density markers specific for indicine breeds were developed by GeneSeek Inc. (Lincoln, NE), but these still suffer from ascertainment bias. However, the whole genome resequencing of single animals can be used to remove that bias, as well as identifying rare putative functional variants and detecting structural variants [10, 12, 17].

In humans, InDels have received far less attention than other variants because they are one of the least well characterized and understood variants across the genome, although several lines of evidence indicated that such variation is a major determinant of human biological diversity [26, 27]. Similarly in cattle, InDels remain less studied than SNVs, despite the fact they are the second most abundant type of genetic variant following SNVs. Although several studies identified a high number of InDels in cattle by next generation sequencing, it is apparent that most of them were not submitted to the dbSNP database. InDels in coding regions can significantly impact gene expression, particularly through frameshifts resulting in prematurely terminated protein products or changed splice variants.

The aim of this study was to detect and make publicly available genome-wide SNVs and InDels present in Gyr, Girolando, Guzerat, and Holstein breeds. These results will contribute to the dbSNP database and will provide an updated genomic resource for genome-wide association studies and genomic selection in indicine cattle, which will help increase knowledge of the underlying genetic architecture of quantitative traits. In addition, our findings could improve comparative genomic studies between *Bos primigenius taurus* and *Bos primigenius indicus* breeds, providing new insights into the history of divergence of those groups.

## Materials and methods

### Ethics statement

Gyr and Guzerat DNA was extracted from semen, while Girolando and Holstein DNA was extracted from the semen or blood samples, bought from an artificial insemination center and therefore, no specific ethical approval is needed (Brazil law number 11794, from October 8th, 2008, Chapter 1, Art. 3, paragraph III). All the samples were obtained with the consent of the artificial insemination center to use for research.

### DNA sampling, library construction and sequencing

A total of 12 animals (two Gyr, three Girolando, two Guzerat and five Holstein) were selected for sequencing according to having high numbers of daughters and due to the role of their lineage within each breed. Gyr and Guzerat genomic DNA was extracted from semen samples using a modified phenol/chloroform method described shortly. Samples were washed with lysis buffer to remove semen diluents. After that, samples were incubated with extraction buffer containing dithiothreitol 10% (DTT) and RNase for 2h. Pellets were incubated overnight with saline-proteinase K buffer and protein was removed by subsequent phenol-chloroform treatment. Girolando (⅝ Holstein and ⅜ Gyr) and Holstein DNA was extracted from the semen or blood samples using the DNeasy Blood & Tissue Kit (Qiagen, Valencia, CA, USA), following manufacturer’s protocol. Quality and concentration of DNA for all samples were determined by NanoDrop 1000 spectrophotometer (Thermo Scientific, Wilmington, DE, USA).

Paired-end and mate-paired libraries with insert sizes of 200 bp (Holstein samples) and 500 bp (Gyr, Guzerat, and Girolando samples) were prepared following Illumina protocols to produces read lengths of 2 x 100 bp and 200 bp. Each library was high-throughput sequenced on a HiSeq 2000 sequencer (Illumina Inc., San Diego, CA, USA), following manufacturer guidelines.

### Short read mapping and variant calling

The UMD 3.1 bovine genome assembly (taurine reference) was used as the reference for the 29 bovine autosomes and the X chromosome whereas the Btau4.6.1 assembly was used as the reference genome sequence for the Y chromosome. Sequencing reads were mapped to the reference assembly using the Burrows-Wheeler Aligner tool (BWA, version 0.7.10-r789) with default parameters [28].

Picard tools (version 1.54) (http://broadinstitute.github.io/picard/) were used to eliminate PCR duplicates. Variant calling was conducted with Freebayes (https://github.com/ekg/freebayes). All SNVs and InDels were identified as differences from the reference genome sequences. The resulting variant list obtained for each animal were filtered by vcffilter (https://github.com/vcflib/vcflib) in order to remove SNVs and InDels with quality scores lower than 30 or coverage lower than 7.

### Annotation of SNVs and InDels

The SNVs and InDels were classified according to their potential function using the Ensembl Variant Effect Predictor tool (VEP, version 84) [29], except those mapped onto Y chromosome because VEP contained only the bovine genome assembly UMD 3.1. Variants with the non-reference allele present in the dbSNP database [30] were classified as “known” and variants described herein for the first time were classified as “novel”. The Venn diagrams representing SNVs and InDels were generated using the R environment (VennDiagram package, version 1.6.17).

The average ratios of homozygous versus heterozygous SNVs and InDels were calculated for Gyr, Girolando and Guzerat breeds, using RTG tools (http://realtimegenomics.com/products/rtg-tools/). The transition-to-transversion ratio (Ti/Tv), which is used as an indicator of potential sequencing errors, was calculated using the vcf-stats tool (http://vcftools.sourceforge.net/perl_module.html#vcf-stats).

### Functional enrichment analysis

The Database for Annotation, Visualization, and Integrated Discovery tool (DAVID, version 6.8) [31, 32] was used for functional enrichment analysis using the lists of genes that had variants classified by the VEP tool as high (splice acceptor variant, splice donor variant, stop gained, frameshift variant, stop lost, and start lost) or moderate (inframe insertion, inframe deletion, missense variant, and protein altering variant) severity of consequences in transcripts. All annotated genes in the taurine genome were used as background. Gene Ontology (GO) biological process, GO cellular component and GO molecular function annotation data sets were used for functional enrichment analysis considering a 10% false discovery rate (FDR) threshold for significance.

### Validation of SNVs using BovineHD BeadChip array

We evaluated the genotype concordance between the SNVs and SNP panel genotype data of Gyr_1, Gyr_2, Girolando_1, Girolando_2, Girolando_3, and Guzerat_2 samples to verify and validate the quality of the identified SNVs. The genotype quality control (QC) was not carried out. A python script was used to compare the SNV calling to the Bovine HD BeadChip array (Illumina, San Diego, USA). The script stored the final report file output from the Illumina Bead Express software used with the BovineHD BeadChip and compared each called locus to its counterpart in the variant calling format (VCF) file containing the SNV calling from the whole-genome sequencing.

## Results and discussion

### Sequencing and variant detection

Approximately 4.3 billion reads were generated with an Illumina HiSeq 2000 sequencer, which for each of seven bulls represented a 10.76 to 16.46-fold coverage of the cattle genome (2,713,722,480 bp of size). A total of 98.78% sequence reads could be mapped to the UMD3.1 bovine genome reference assembly (Table 1). Sequencing at 15-fold coverage of the genome has been reported to enable identification of around 75% of heterozygote variants and an increase in sequence depth significantly improves the accuracy and sensitivity of variant identification [33].

**Table 1:** Summary of sequencing and assembly results for the seven bulls representing Gyr, Girolando, Guzerat and Holstein cattle breeds.

Sample	Total reads	Mapped reads	Coverage
Gyr_1	407,432,426	98.98%	15.01
Gyr_2	420,236,440	98.75%	15.49
Girolando_1	297,636,324	99.07%	10.97
Girolando_2	446,757,536	98.90%	16.46
Girolando_3	345,972,522	99.05%	12.75
Guzerat_1	352,056,808	98.97%	12.97
Guzerat_2	445,595,922	98.88%	16.42
Holstein_1	338,028,986	99.02%	12.46
Holstein_2	326,956,582	99.71%	12.05
Holstein_3	380,512,652	99.60%	14.02
Holstein_4	291,946,026	99.71%	10.76
Holstein_5	304,111,802	94.35%	11.21

A total of 27.441.279 SNVs (58.09% from Gyr, 48.42% from Girolando, 61.01% from Guzerat, and 32.59% from Holstein) were identified in the samples (Table 2), of which 2,557,670 (37.96% Gyr, 24.51% Girolando, 34.96% Guzerat, and 24.48% Holstein) were novel, while the remainder of the SNVs had been previously uploaded to the dbSNP database. Given the large number of novel variants, these results indicate that a high number of genetic variants remain to be identified in the bovine genome. It is important to note that the lower proportion of novel variants discovered in the Holstein breed was expected, because its genome has already been extensively re-sequenced [10, 12, 17, 34].

**Table 2:** Summary of SNVs and InDels identified in this study for Gyr, Girolando, Guzerat and Holstein breeds.

Breed	SNVs		InDels	
	total	novel	total	novel
Gyr	15,941,804	970,823	1,833,387	342,899
Girolando	13,286,669	626,921	1,413,047	227,176
Guzerat	16,743,392	894,177	1,975,563	370,095
Holstein	8,944,009	626,269	1,348,564	228,835

In recent years, the improvement of high-throughput sequencing platforms and sequence analysis tools has facilitated the identification and characterization of genetic variants in many cattle breeds, increasing the number of variants available in the dbSNP. Liao et al. [20] re-sequenced 14 Gyr animals and identified around ten million SNPs and six hundred thousand InDels, of which 62.34% and 83.62%, respectively, were novel at that time. In this study, we also identified a high number of variants in Gyr breed, of which 6.09% of SNVs and 18.70% of InDels were novel according to the dbSNP.

A total of 5,198,306 (32.61%) SNVs were common across the two Gyr samples, 1,268,464 (9.55%) common across three Girolando samples, 7,010,781 (41.87%) common across two Guzerat samples, and 590,785 (6.60%) common across five Holstein DNA samples. A total of 3,828,041 InDels (47.89% Gyr, 36.91% Girolando, 51.61% Guzerat, and 35.23% Holstein) were identified, of which 883,219 (38.82% Gyr, 25.72% Girolando, 41.90% Guzerat, and 25.91% Holstein) were novel (Table 2).

A total of 430,501 (23.48%) InDels were common across Gyr, 85,638 (6.06%) common across Girolando, 585,825 (29.65%) common across Guzerat, and 72,103 (5.35%) common across Holstein samples. The high number of unique genetic variants identified for each animal within the same breed shows the importance of re-sequencing to identify novel variants for monitoring genetic diversity in the cattle breeds and for developing strategies to prevent some eventual loss of genetic variability.

**Fig 1:**
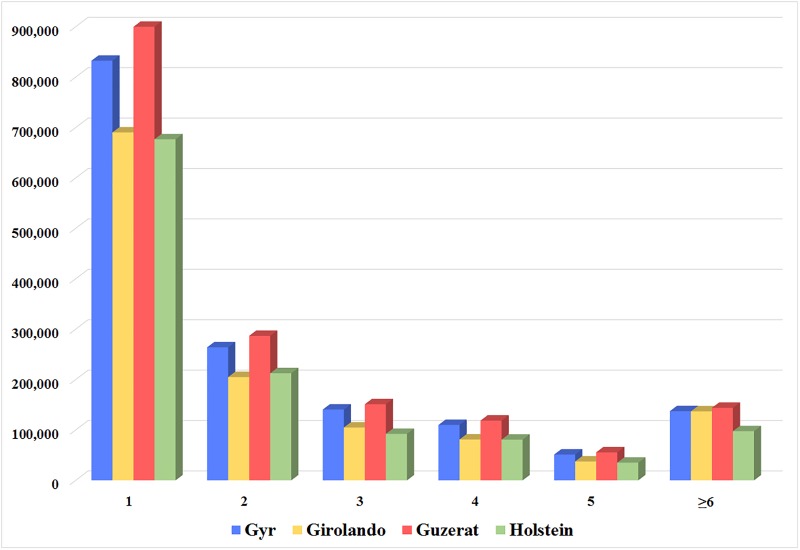
Distribution of InDel lengths (bp) for Gyr, Girolando, Guzerat, and Holstein cattle breeds.

The length of InDels ranged from -44 bp (deletion) to +30 (insertion) bp in Holstein breed, -43 bp (deletion) to +29 bp (insertion) in Gyr breed, and -43 bp (deletion) to +29 bp (insertion) for Guzerat and Girolando breeds. Moreover, most of the identified InDels have lengths less than 4 bp (87.72% Gyr, 86.10% Girolando, 87.94% Guzerat and 88.89% Holstein InDels) (Fig 1).

The number of deletions discovered was slightly lower than the number of insertions, demonstrated by insertion/deletion ratios (Table 3). The higher average ratio of heterozygous versus homozygous SNVs and InDels observed in Girolando breed (Table 3) can be explained by its composite breed makeup, since it comprises admixed Holstein (taurine) and Gyr (indicine) genomes.

**Table 3:** Description of insertion-to-deletion, heterozygous-to-homozygous (Het/Hom) and transition-to-transversion (Ti/Tv) ratios for InDels and SNVs.

Sample	Insertions/Deletions					SNVs	
	Insertion/Deletion	Insertion Het/Hom	Deletion Het/Hom	InDel Het/Hom	Total Het/Hom	Ti/Tv	Het/Hom
Gyr_1	0.94	1.05	1.38	1.70	1.26	2.33	1.23
Gyr_2	0.92	1.08	1.42	1.66	1.37	2.37	1.34
Girolando_1	0.96	2.47	3.58	4.26	3.74	2.37	3.73
Girolando_2	0.94	2.49	3.51	3.78	4.01	2.30	4.02
Girolando_3	0.97	1.91	2.56	3.15	2.65	2.28	2.65
Guzerat_1	0.95	1.09	1.46	1.84	1.32	2.34	1.29
Guzerat_2	0.94	1.19	1.54	1.92	1.39	2.33	1.35
Holstein_1	0.98	1.34	1.63	2.06	1.68	2.06	1.68
Holstein_2	0.99	1.37	1.71	2.11	1.76	2.12	1.76
Holstein_3	0.98	1.37	1.71	2.05	1.79	2.16	1.79
Holstein_4	1.00	1.49	1.91	2.33	1.89	2.12	1.88
Holstein_5	0.96	0.85	0.94	1.19	0.95	1.64	0.93

The transition-to-transversion (Ti/Tv) ratio was calculated for each sample (Table 3) as an indicator of potential random sequencing errors. The Ti/Tv average ratio was 2.20 for all samples, which indicated a high accuracy for most of the variants identified in this study. Our results are supported by previous studies in bovine, which found Ti/Tv ratios of approximately 2.2 in Hanwoo, Yanbian, Jeju Heugu, Goldwyn, Black Angus, and Holstein breeds [17, 18, 35].

All the SNVs and InDels identified in this study were submitted in variant calling format (vcf) to the dbSNP database under the handle name EMBRAPA-CNPGL-LBGA.

The density of variants within each chromosome was proportional to chromosome length, except for the X and Y chromosomes which showed a low number of variants identified (S1 Table). These findings were expected because sex chromosomes are haploid in males, resulting in a sequencing depth about half that of the autosomes and, as consequence, a lower rate of variant identification [15, 16]. In addition, some studies have suggested that selection resulted in lower observed retention of mutant variants on genes located on sex chromosomes because of the effects of exposure to deleterious recessive alleles in hemizygous conditions [15, 36, 37].

**Fig 2:**
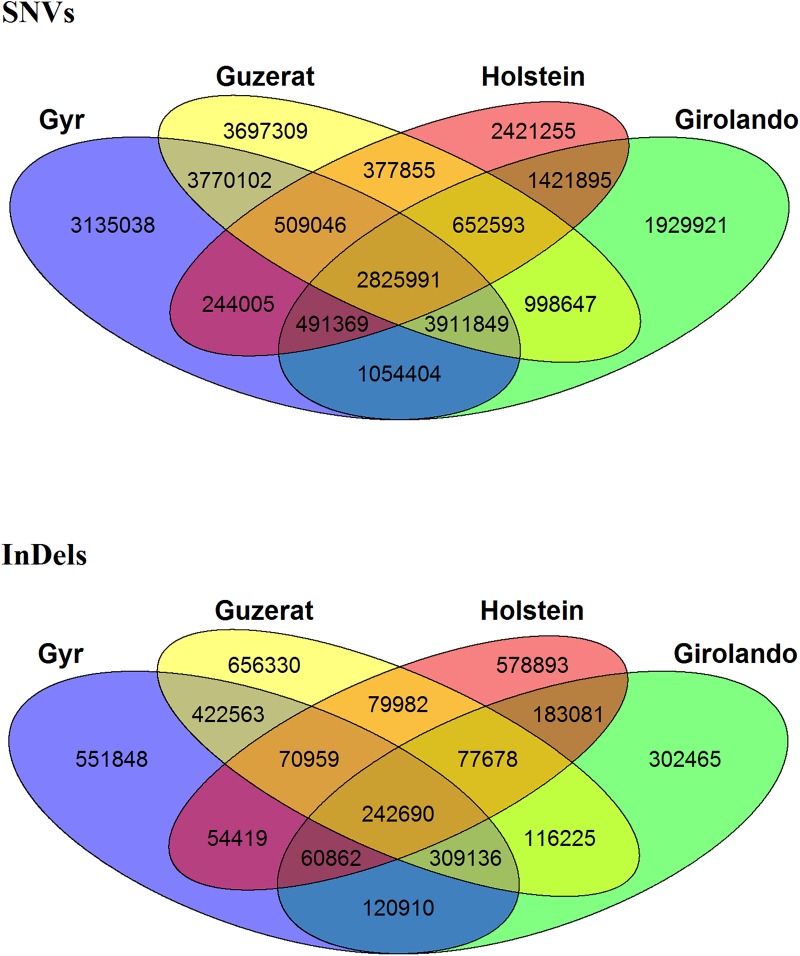
Venn diagram showing the overlap of all identified SNVs and InDels in the Gyr, Girolando, Guzerat, and Holstein genomes.

A high number of SNVs and InDels were identified as being shared among indicine breeds (Fig 2). A total of 2,825,991 (10.30%) SNVs were common to all four breeds while the SNVs with no overlap with any other breed (i.e. breed-specific) represented 11.42% in Gyr, 7.03% in Girolando, 13.47% in Guzerat, and 8.82% in Holstein breeds. The number of InDels shared among all four breeds was 242,690 (6.34%), while the proportion of breed-specific InDels was 14.41% in Gyr, 7.90% in Girolando, 17.14% in Guzerat, and 15.12% in Holstein (Fig 2).

Breed-specific variants could be useful for further studies about breed characterization, while overlapping variants may reflect closer relations between breeds. Although the Gyr and Holstein breeds were used to develop the Girolando composite breed (⅝ Holstein and ⅜ Gyr), we identified a closer overlap of variants common to Gyr and Guzerat (11,016,988 SNVs and 1,045,348 InDels), both indicine breeds than those common to Girolando and Gyr (8,236,613 SNVs and 733,598 InDels) or to Girolando and Holstein (5,391,848 SNVs and 564,311 InDels) breeds. Also, the number of genetic variants shared among Girolando, Gyr, and Holstein breeds reflect the current genetic state of this Girolando population, which appears to be approximately ⅝ Gyr and ⅜ Holstein. Apparently, the Girolando breed composition is changing since the breed was formed and the selection pressure has keeping the most variants for Gyr breed in this environment, maybe due to its additional advantages in tropical climates when compared to Holsteins breed, such as adaptability to high-temperature conditions and resistance to parasites.

### Annotation of SNVs and InDels

Identified SNVs and InDels (Table 4) were annotated into functional categories using the VEP tool, except those that were mapped on the Y chromosome (S2 Table). Variant annotation indicated that variants may be functionally relevant for each breed. The number of variant annotations was higher than the number of SNVs and InDels identified, because the same genetic variant can have more than one annotation (Table 4).

**Table 4:** Summary of functional classification of SNVs and InDels identified in Gyr, Girolando, Guzerat and Holstein breeds. Some SNVs and InDels are represented in multiple categories.

Classification	Gyr	Girolando	Guzerat	Holstein
Splice acceptor variant	385	348	376	276
Splice donor variant	368	346	370	225
Stop gained	535	481	598	534
Frameshift variant	1,479	1,364	1,491	987
Stop lost	47	35	42	27
Start lost	107	100	102	56
Inframe insertion	277	235	238	92
Inframe deletion	358	306	302	153
Missense variant	53,519	48,073	54,433	26,931
Protein altering variant	74	72	66	50
Splice region variant	15,337	12,870	15,172	6,620
Synonymous variant	78,030	66,437	77,527	27,820
Stop retained variant	52	46	51	26
Coding sequence variant	396	360	366	221
Mature miRNA variant	120	111	132	81
5_prime_UTR_variant	8,643	7,518	7,945	2,245
3_prime_UTR_variant	42,266	36,082	42,233	19,609
Non-coding transcript exon variant	8,260	7,019	8,610	4,523
Intron variant	5,648,353	4,701,181	5,845,387	2,956,134
Non-coding transcript variant	8,628	7,368	8,979	4,710
Upstream gene variant	893,243	765,141	892,485	458,765
Downstream gene variant	899,298	766,050	910,644	457,038
Intergenic variant	12,442,695	10,192,691	13,244,468	7,556,938

Most variants were identified in intergenic or intronic, while a small number of variants were annotated as being in genic regions including exons, untranslated regions (5’UTR and 3’UTR) and splice sites (Table 4). A significant number of variants were predicted to be deleterious in Gyr (7,654), Girolando (7,022), Guzerat (7,743), and Holstein (4,699) cattle breeds.

In regard to variants annotated on the Y chromosome (S2 Table), most variants were mapped to intergenic regions (87.31% Gyr, 88.34% Girolando, 88.42% Guzerat, and 92% Holstein), while a small number of variants were annotated in genic regions, mainly in coding regions (0.71% Gyr, 0.63% Girolando, 0.47% Guzerat, and 0.13% Holstein). This lower representation in coding regions for the Y chromosome compared to the corresponding representation in autosomal variants only reflects the fewer number of genes present on the Y when compared to autosomes. The identification of variants in genes from the Y chromosome is important for breeding programs, since these could be responsible for fertility-related traits [38].

### Functional enrichment analysis

Genetic variants classified by the VEP tool as high (disruptive impact in the protein causing protein truncation, loss of function or triggering nonsense mediated decay) or moderate (non-disruptive variant that could change protein effectiveness) severity consequences in transcripts provide a useful resource to be used in genetic analysis of phenotypic differences observed among these four breeds, mainly because these types of variants can influence economically important traits. For this reason, following variant annotation, functional enrichment analysis was performed using the DAVID tool. The analysis set comprised 11,940 Ensembl IDs from Gyr, 11,268 from Girolando, 11,910 from Guzerat and 7,617 from Holstein. Gene Ontology enrichment analysis revealed that three GO biological processes (GO:0007186~G-protein coupled receptor signaling pathway, GO:0050907~detection of chemical stimulus involved in sensory perception, and GO:0007608~sensory perception of smell), five GO molecular functions (GO:0004984~olfactory receptor activity, GO:0004930~G-protein coupled receptor activity, GO:0004888~transmembrane signaling receptor activity, GO:0005549~odorant binding, and GO:0005524~ATP binding) and two GO cellular components (GO:0016021~integral component of membrane and GO:0005886~plasma membrane) were enriched in all four breeds studied, considering a 10% FDR threshold for significance (Table 5).

**Table 5:** Gene Ontology (GO) terms and KEGG pathways enriched (FDR<0.10) in Gyr, Girolando, Guzerat and Holstein breeds.

Terms	Gyr		Girolando		Guzerat		Holstein	
	Genes	FDR	Genes	FDR	Genes	FDR	Genes	FDR
***GO Biological Process***								
GO:0007186 G-protein coupled receptor signaling pathway	612	5.35E-25	615	6.38E-37	632	4.24E-34	531	7.81E-63
GO:0007608 Sensory perception of smell	173	1.24E-09	173	6.66E-13	182	1.47E-15	153	1.01E-21
GO:0050907 Detection of chemical stimulus involved in sensory perception	135	1.84E-09	135	2.88E-12	137	7.68E-11	124	3.30E-22
GO:0050911 Detection of chemical stimulus involved in sensory perception of smell	-	-	45	0.03039	46	0.05485	45	8.99E-09
GO:0006508 Proteolysis	139	0.02278	-	-	-	-	-	-
GO:0007165 Signal transduction	297	0.02108	-	-	-	-	207	0.01465
***GO Cellular Component***								
GO:0016021 Integral component of membrane	2878	2.32E-11	2736	5.33E-13	2874	5.97E-11	1929	3.42E-15
GO:0005886 Plasma membrane	1643	1.17E-18	1618	2.55E-31	1680	5.21E-27	1192	6.07E-34
GO:0005887 Integral component of plasma membrane	589	0.00130	-	-	-	-	-	-
GO:0045095 Keratin filament	-	-	48	0.02374	-	-	-	-
***GO Molecular Function***								
GO:0004984 Olfactory receptor activity	661	2.86E-36	681	3.35E-59	694	7.46E-55	613	1.51E-102
GO:0004930 G-protein coupled receptor activity	764	5.20E-32	775	9.03E-49	796	1.60E-46	672	3.52E-83
GO:0004888 Transmembrane signaling receptor activity	165	3.78E-11	163	1.19E-12	167	2.98E-12	144	1.48E-20
GO:0005549 Odorant binding	143	3.36E-07	143	1.68E-09	145	3.69E-08	125	8.74E-16
GO:0005524 ATP binding	871	6.08E-05	812	0.05890	880	5.89E-06	621	4.21E-09
GO:0042626 ATPase activity, coupled to transmembrane movement of substances	-	-	-	-	-	-	36	0.00680
***KEGG Pathway***								
bta04740 Olfactory transduction	705	0.00217	723	7.39E-12	736	7.06E-09	656	1.51E-50
bta04512 ECM-receptor interaction	-	-	68	0.10062	71	0.04028	-	-
bta02010 ABC transporters	-	-	-	-	-	-	33	0.00254
bta01100 Metabolic pathways	812	0.02570	-	-	-	-	-	-

Four KEGG (Kyoto Encyclopedia of Genes and Genomes) pathways were identified as being over represented by DAVID. The olfactory transduction pathway (bta04740) was over represented in all four cattle breeds, the ECM-receptor interaction pathway (bta04512) was over represented in Girolando and Guzerat breeds, while the ABC transporters (bta02010) and the metabolic pathways (bta01100) were over represented only in Holstein and Gyr, respectively (Table 5).

Olfactory transduction pathways act in the perception of odor through olfactory receptors and biochemical signaling events, which influences food preference and food consumption [39]. Zhan et al. [15] identified enrichment bias for this pathway when re-sequencing a Holstein Friesian bull and performing an enrichment analysis for genes with nonsynonymous SNVs. Do et al. [40] reported that the olfactory transduction pathway is associated with residual feed intake in pigs. Although the novel variants identified in this study were spread throughout the genome, we identified a high number of novel variants clustered on genes from BTA12 and BTA15 in all four cattle breeds, which could be explained by the presence of gene families that creates problems in the mapping, resulting in erroneous identification of genetic variation within these related genes [12]. The olfactory receptor genes comprise the largest multigene family in vertebrates [41], with more than one thousand coding genes organized in clusters on 26 cattle chromosomes, of which BTA15 has the largest number of mapped olfactory receptor functional genes (n = 251) [42]. The genes from BTA12 enriched with novel variants comprised multidrug resistance-associated protein 4 genes (*LOC100299180*, *LOC509854*, *LOC515333*, *LOC522174*, *LOC523126*, and *LOC53043*), while BTA15 showed the olfactory receptor genes (*LOC100299084*, *LOC100336901*, *LOC104968619*, *LOC508180*, *LOC509025*, *LOC521081*, *LOC615051*, *LOC787385*, *LOC787428*, *LOC789288*, *LOC789293*, *LOC789300*, *LOC790274*, *LOC100299808*, *OR4C15*, *OR5F1*, and *OR8K1*) contained a large number of novel variants.

The extracellular matrix (ECM) consists of a complex of structural and functional macromolecules with an important role in tissue and organ morphogenesis and in the maintenance of cell and tissue structure and function. It can directly or indirectly influence specific cellular activities such as differentiation, adhesion, proliferation, and migration. Lee et al. [43] identified the ECM-receptor interaction pathway enriched in expression analyses from omental, subcutaneous and intramuscular adipose tissues in cattle, acting also as a sub-pathway of other enriched pathways. Gao et al. [44] reported that the ECM-receptor interaction pathway is transcriptionally regulated throughout the onset of lactation in Holstein cows.

The ATP-binding cassette (ABC) transporters is one of the largest protein families in prokaryotes and eukaryotes. They couple ATP hydrolysis to active transport of a wide variety of substrates such as sugars, peptides, proteins, lipids, sterols, ions, and drugs. Prokaryotic antibiotic resistance and eukaryotic multi-drug resistance is associated with ABC transporters, which acts as a modulator of drug absorption and distribution [45, 46].

Over-represented KEGG pathways and GO terms and results should be carefully interpreted because of the small sample size used in this study. However, these results provide important genomic information to investigate the genetic mechanisms underlying phenotypic differences and similarities among these breeds.

### Validation of SNVs using Bovine HD BeadChip array

The quality of SNVs identified in this study was evaluated by comparison of the concordance between the genotypes obtained from Illumina BovineHD BeadChip array for the Gyr_1, Gyr_2, Girolando_1, Girolando_2, Girolando_3, and Guzerat_2 samples. Around 98.46–99.45% of detectable array SNPs were identified as SNV by sequencing, which indicated that most of the SNVs were correctly called in this study. Table 6 shows the percentage of SNVs from the Bovine HD BeadChip array that are also present in the SNV calling and the concordance between array and whole-genome sequence genotypes.

**Table 6:** Comparison of BovineHD BeadChip array genotypes to sequencing SNVs.

Sample	Sequencing calls*	Concordant	Discordant
Gyr_1	99.42%	267,258	1,558
Gyr_2	99.45%	196,537	1,091
Girolando_1	98.46%	145,365	2,274
Girolando_2	98.92%	153,157	1,670
Girolando_3	98.66%	185,638	2,527
Guzerat_2	99.39%	247,931	1,526

*Percentage of detectable array SNPs that were identified as SNV by sequencing

## Conclusion

In this study, we presented an extensive genome analysis of Gyr, Girolando, Guzerat and Holstein breeds following whole-genome re-sequencing using an Illumina HiSeq 2000 sequencing platform, which lead to identification of 27,441,279 SNVs and 3,828,041InDels in the cattle genome. The high number of genetic variants identified for each breed is important to monitoring genetic diversity and for developing strategies to prevent an eventual loss of genetic variability. The submission of the approximately 3.4 million novel variants to the dbSNP has significantly increased the number of variants available, particularly for the *Bos primigenius indicus* genome. The annotation of SNVs InDels predicted to affect function of transcripts reveal variants that could contribute to phenotypic differences among the Gyr, Girolando, Guzerat and Holstein breeds. Also, we identified changes in the Girolando breed genetic composition since it was formed, which suggests that the selection pressure has preferred to retain the variants for Gyr breed in the tropical climates, maybe due to their particular advantages in this environment when compared to Holstein, such as rusticity, thermotolerance, and resistance to endoparasites and ectoparasites. Therefore, this study provides the basis for further investigations about potential genomic markers and molecular mechanisms associated with economically important traits for breeding programs of these cattle breeds.

## Supporting information

S1 Table Summary of variants identified in each breed grouped by chromosome. (DOCX)

S2 Table Summary of SNVs and InDels mapped on the bovine Y chromosome. (DOCX)
