# Supplementary material for: Single nucleotide variants and InDels identified from whole-genome re-sequencing of Guzerat, Gyr, Girolando and Holstein cattle breeds
Source: PLoS One. 2017 Mar 21;12(3):e0173954. doi: 10.1371/journal.pone.0173954 (PMC5360315; doi:10.1371/journal.pone.0173954)
Supplement: S1 Table — (DOCX) [file pone.0173954.s001.docx]

**S1 Table. Summary of variants identified in each breed grouped by chromosome.**

| **BTA** | **SNVs** | | | | | | |  | **InDels** | | |
| --- | --- | --- | --- | --- | --- | --- | --- | --- | --- | --- | --- |
|  | **Gyr** | **Girolando** | **Guzerat** | **Holstein** |  | **Gyr** | **Girolando** | | | **Guzerat** | **Holstein** |
| 1 | 953,906 | 746,157 | 1,017,539 | 564,777 |  | 108,071 | 78,118 | | | 120,258 | 88,141 |
| 2 | 768,600 | 688,656 | 822,059 | 453,717 |  | 88,150 | 72,567 | | | 97,890 | 69,859 |
| 3 | 689,964 | 606,098 | 724,004 | 389,777 |  | 77,362 | 62,139 | | | 83,842 | 56,512 |
| 4 | 767,754 | 572,709 | 815,604 | 436,615 |  | 90,055 | 61,704 | | | 99,249 | 67,692 |
| 5 | 693,827 | 682,814 | 749,218 | 408,216 |  | 79,761 | 71,655 | | | 88,373 | 61,517 |
| 6 | 698,904 | 688,212 | 755,945 | 430,600 |  | 78,799 | 72,517 | | | 89,032 | 67,764 |
| 7 | 648,271 | 560,365 | 683,528 | 363,904 |  | 75,951 | 60,338 | | | 82,270 | 55,930 |
| 8 | 671,916 | 503,153 | 720,421 | 374,092 |  | 76,790 | 52,515 | | | 85,637 | 56,946 |
| 9 | 603,959 | 448,603 | 653,137 | 379,176 |  | 69,585 | 48,431 | | | 78,361 | 59,337 |
| 10 | 642,873 | 500,258 | 648,069 | 353,265 |  | 74,507 | 53,536 | | | 77,832 | 52,331 |
| 11 | 648,816 | 580,476 | 675,302 | 348,561 |  | 73,434 | 60,215 | | | 77,765 | 51,935 |
| 12 | 618,888 | 547,859 | 678,845 | 440,334 |  | 72,166 | 59,751 | | | 83,435 | 70,030 |
| 13 | 526,419 | 438,338 | 532,390 | 256,811 |  | 61,977 | 47,014 | | | 63,581 | 37,382 |
| 14 | 507,271 | 486,776 | 533,825 | 276,781 |  | 57,827 | 52,627 | | | 62,349 | 41,520 |
| 15 | 568,441 | 388,098 | 608,635 | 325,049 |  | 65,828 | 41,384 | | | 71,557 | 48,896 |
| 16 | 506,691 | 254,172 | 547,816 | 272,807 |  | 58,135 | 28,811 | | | 64,124 | 40,533 |
| 17 | 475,057 | 452,855 | 485,272 | 252,806 |  | 56,068 | 50,064 | | | 58,294 | 39,004 |
| 18 | 424,859 | 279,026 | 433,917 | 204,935 |  | 50,884 | 31,016 | | | 51,004 | 29,426 |
| 19 | 398,817 | 381,587 | 406,774 | 185,463 |  | 47,787 | 42,176 | | | 47,747 | 27,204 |
| 20 | 433,001 | 313,564 | 482,712 | 260,899 |  | 48,782 | 32,714 | | | 57,468 | 39,959 |
| 21 | 468,099 | 442,910 | 480,959 | 235,130 |  | 54,327 | 47,318 | | | 55,991 | 34,936 |
| 22 | 384,577 | 313,389 | 377,127 | 173,784 |  | 45,003 | 33,713 | | | 44,699 | 26,210 |
| 23 | 370,379 | 328,187 | 403,434 | 229,390 |  | 44,289 | 36,410 | | | 48,353 | 33,497 |
| 24 | 425,283 | 383,549 | 439,483 | 211,745 |  | 49,658 | 40,784 | | | 51,333 | 31,862 |
| 25 | 289,716 | 284,454 | 287,692 | 133,231 |  | 33,295 | 29,835 | | | 32,403 | 18,027 |
| 26 | 343,307 | 278,388 | 341,114 | 172,245 |  | 39,532 | 29,212 | | | 40,149 | 25,687 |
| 27 | 328,891 | 255,942 | 325,745 | 184,122 |  | 39,296 | 28,107 | | | 39,538 | 27,490 |
| 28 | 315,823 | 264,130 | 344,996 | 164,150 |  | 36,274 | 27,678 | | | 40,725 | 23,530 |
| 29 | 366,321 | 303,446 | 385,037 | 204,233 |  | 41,324 | 31,704 | | | 43,963 | 28,759 |
| X | 303,612 | 224,342 | 287,095 | 193,493 |  | 29,447 | 21,446 | | | 28,841 | 29,861 |
| Y | 5,881 | 1,841 | 5,739 | 1,932 |  | 616 | 220 | | | 657 | 297 |
| MT | 30 | 134 | 195 | 280 |  | 4 | 23 | | | 36 | 47 |
| Unknown | 91,651 | 86,181 | 89,764 | 61,689 |  | 8,403 | 7,305 | | | 8,807 | 6,443 |
| **Total** | **15,941,804** | **13,286,669** | **16,743,392** | **8,944,009** |  | **1,833,387** | **1,413,047** | | | **1,975,563** | **1,348,564** |
