# Supplementary material for: Single nucleotide variants and InDels identified from whole-genome re-sequencing of Guzerat, Gyr, Girolando and Holstein cattle breeds
Source: PLoS One. 2017 Mar 21;12(3):e0173954. doi: 10.1371/journal.pone.0173954 (PMC5360315; doi:10.1371/journal.pone.0173954)
Supplement: S2 Table — (DOCX) [file pone.0173954.s002.docx]

**S2 Table. Summary of SNVs and InDels mapped on the bovine Y chromosome.**

| **Breed** | **Mapped** | **Exon** | **Intergenic** | **Intron** | **Other** |
| --- | --- | --- | --- | --- | --- |
| ***SNVs*** |  |  |  |  |  |
| Gyr | 5,880 | 36 | 5,121 | 560 | 163 |
| Girolando | 1,840 | 13 | 1,610 | 172 | 45 |
| Guzerat | 5,738 | 24 | 5,065 | 466 | 183 |
| Holstein | 1,931 | 2 | 1,772 | 118 | 39 |
| ***InDels*** |  |  |  |  |  |
| Gyr | 615 | 10 | 550 | 45 | 10 |
| Girolando | 219 | 0 | 209 | 9 | 1 |
| Guzerat | 656 | 6 | 589 | 47 | 14 |
| Holstein | 296 | 1 | 277 | 10 | 8 |
